# Acknowledgment of Reviewers

**DOI:** 10.1128/mBio.02859-18

**Published:** 2019-02-05

**Authors:** Arturo Casadevall

**Affiliations:** a Bloomberg School of Public Health, Johns Hopkins University, Baltimore, Maryland, USA

## EDITORIAL

On behalf of the editors of *mBio*, I gratefully acknowledge the following individuals, who served as reviewers for the journal in 2018. Their time and effort in reviewing articles have been essential to ensuring the high quality of our publications, and their help is greatly appreciated.

Hans Ackerman
Margaret E. Ackerman
Karen Adair
Abigail A. Adebusuyi
Abram Aertsen
Luis M. Agosto
Hector C. Aguilar
Jinwoo Ahn
Tero Ahola
Baki Akgül
Serap Aksoy
Gladys Alexandre
Francis Alonzo III
Sam Alsford
Friedrich Altmann
Pedro M. Alzari
Rama Rao Amara
Zandrea Ambrose
Jorge Amich
Pascal Amireault
Zhiqiang An
Karthik Anantharaman
Dan I. Andersson
Hiroyuki Arai
Cesar A. Arias
Chelsie Elizabeth Armbruster
Michelle M. Arnold
Philip M. Ashton
John Atkins
Shannon Wing-Ngor Au
Gordon Aubree
Mes Ayrapetyan
Paul Babitzke
Subash Babu
Michael A. Bachman
Steffen Backert
Nathalie Balaban
Jen Balkus
Elizabeth Ballou
Bruce W. Banfield
Ehud Banin
Lawrence Banks
Fernando Baquero
Rodolphe Barrangou
Alan D. Barrett
Jeffrey E. Barrick
Erik S. Barton
Randall J. Basaraba
Paul Bates
Florian F. Bauer
Amy Baxter
Bernard W. Beall
Bruno Beaumelle
Pascale B. Beauregard
Morgan Beeby
Michael Behnke
Marcel A. Behr
Chase Beisel
Faith C. Belanger
George A. Belov
Clara Belzer
Jose A. Bengoechea
Thomas Ulrich Berendonk
Teresa M. Bergholz
Ben Berkhout
Thomas Bernhardt
Timothy Bestor
Devaki Bhaya
Marco Biddau
Amy Biddle
Elaine Bignell
Russell E. Bishop
Dieter Blaas
Ira J. Blader
Carol D. Blair
David Blair
James B. Bliska
Jesse D. Bloom
Helge B. Bode
Ivo G. Boneca
Paola Bonfante
Seth Bordenstein
Katherine A. Borkovich
Helena Ingrid Boshoff
Tolga Bozkurt
Axel Brakhage
Catherine Braun-Breton
Erhard Bremer
Ariane Briegel
Thomas Briese
Volker Briken
Shaun R. Brinsmade
William J. Britt
Robert A. Britton
Michael A. Brockhurst
Michael John Bromley
Roland Brosch
Daren W. Brown
Sam Brown
John H. Brumell
Yves V. Brun
Caroline Brunel
Donald A. Bryant
Juliane Bubeck Wardenburg
Carmen Buchrieser
Angus Buckling
Nienke Buddelmeijer
Bernd Bukau
Timothy Bullock
Per Bullough
Kristin M. Burkholder
Michael B. Burns
Briana M. Burton
Jeremy P. Burton
Geraldine Butler
Julea N. Butt
Michael J. Calcutt
Andrew Camilli
Gabriella Campadelli-Fiume
Kenneth Campellone
David Cánovas
Valerie Jean Carabetta
Nicolas Carraro
Jim Cassat
Amanda Casto
Clayton C. Caswell
Liraz Chai
Yunrong Chai
Henry F. Chambers
Patricia Ann Champion
Hubert Charles
Trevor Charles
Linda Chelico
Liang Chen
Mingzhou Chen
Swaine L. Chen
Xian-Ming Chen
Yan Ping Chen
Yin Chen
Ambrose L. Cheung
Ludmila Chistoserdova
Chetan E. Chitnis
Hyeryun Choe
Yit Heng Chooi
Hisn-Hung (David) Chou
Taniya Roy Chowdhury
Cornelius Clancy
Gerard Clarke
Christine Clayton
Phillip S. Coburn
Frederick M. Cohan
Taylor S. Cohen
Maria Carmen Collado
Jérôme Collemare
Bianca Colonna
Laurie E. Comstock
Gregory M. Cook
Vaughn S. Cooper
Teresa M. Coque
Pascale Cossart
Marcio Costa
Peggy A. Cotter
Patrice Courvalin
Jeffery S. Cox
Laura M. Cox
Brendan Crabb
Robert A. Cramer
Sue Ellen Crawford
Sean Crosson
Nicholas J. Croucher
Colin Crump
Roy Curtiss III
Melanie T. Cushion
Ross E. Dalbey
Zachary D. Dalebroux
Blossom Damania
Ajai A. Dandekar
Pranav Danthi
Andrew J. Darwin
Andrew D. Davidson
Bryan William Davies
Andrew J. Davison
Kasey Day
Marina de Bernard
William F. DeGrado
Kirk W. Deitsch
Tania F. de Koning-Ward
Miguel A. de la Cruz
Juan Carlos de la Torre
Nicholas R. De Lay
Maurizio Del Poeta
Vincent J. Denef
Hilde De Reuse
Petra Dersch
Rik L. de Swart
Floyd E. Dewhirst
Y. Peter Di
George Diallinas
Gill Diamond
Samuel L. Díaz-Muñoz
Johan Dicksved
Stephen P. Diggle
Alan Angelo DiSpirito
Maziar Divangahi
Thuy Do
Leslie L. Domier
Samuel Dominguez
Anna Dongari-Bagtzoglou
Curtis Donskey
Tobias Dörr
Ivan D'Orso
Angela E. Douglas
Simon L. Dove
Shaynoor Dramsi
Liangcheng Du
Rebbeca Duar
David Dubnau
John Dunbar
Jay Dunlap
Mary Dunlop
W. Paul Duprex
Jonathan Dworkin
Leo Eberl
Jonathan Eisen
Craig D. Ellermeier
Gillian Elliott
David Enard
Tobias Engl
Lynn W. Enquist
Aaron Conrad Ericsson
Olivier Espeli
Eduardo A. Espeso
Matthew J. Evans
Sarah Elisabeth Ewald
Federica Facciotti
Robert Fagan
Joseph O. Falkinham
John T. Fallon
Lu Fan
Ferric C. Fang
Georg Fantner
Michael Farzan
Rachel Fearns
Nicholas Feasey
Mario Feldman
Qin Feng
Zongdi Feng
Paul D. Fey
David A. Fidock
Aretha Fiebig
Sergio R. Filipe
Scott G. Filler
Sabine Fillinger
Andrés Finzi
PierLuigi Fiori
Vincent A. Fischetti
Kathryn Fixen
Suzanne M. J. Fleiszig
Antje Flieger
Sabine Flitsch
Ervin Fodor
James Craig Forrest
Kevin Foster
Simon J. Foster
Timothy J. Foster
Betsy Foxman
Olivera Francetic
Cornet Francois
Martin Fraunholz
Stephen J. Free
Eric O. Freed
Dorte Frees
Martyn A. French
Matthew B. Frieman
Paul D. Friesen
Teresa Frisan
Thilo Fuchs
Clay Fuqua
Michael Gale
Sheetal Gandotra
Barbie K. Ganser-Pornillos
Haichun Gao
Shou-Jiang Gao
Francisco García-del Portillo
Ferran Garcia-Pichel
Rozenn Gardan
Kevin W. Garey
Ethan Garner
fabien Garnier
Ricardo T. Gazzinelli
Angie Gelli
Pierre Genevaux
Ralf Gerhard
Johannes Gescher
Jean-Marc Ghigo
Sean M. Gibbons
Karine A Gibbs
David P. Giedroc
Tim Wolf Gilberger
Jason J. Gill
Steven R. Gill
Michael Gillings
Brendan F. Gilmore
Pierre Gladieux
Philippe Glaser
Jennifer B. Glass
N. Louise Glass
Erin Goley
Ivo Gomperts Boneca
Bertha Gonzalez-Pedrajo
Felicia Goodrum
Erica M. Goss
Paul Gottlieb
Friedrich Götz
Celia Goulding
Mark Goulian
Revathi Govind
Elena A. Govorkova
Neil A. Gow
Jeffrey A. Gralnick
Elisa Teresa Granato
Christophe Grangasse
Andrew J. Grant
Edward M. Greenfield
Simonetta Gribaldo
Hans-Peter F. Grossart
Anne Grove
Angelika Gründling
Tingyue Gu
Marc-Jan Gubbels
Christophe Guilhot
Georg Häcker
Maria Hadjifrangiskou
Alex Hall
Larry J. Halverson
Brian K. Hammer
Neal D. Hammer
Kaphoon Han
Cara H. Haney
Siegfried Hapfelmeier
Jens Harder
Gail G. Hardy
Richard Harrigan
Rasika Harshey
Graham F. Hatfull
Roland Hatzenpichler
Vasili Hauryliuk
Philippe Marcel Hauser
Robert P. Hausinger
Ya-Wen He
Patrick Hearing
Brian P. Hedlund
Richard G. Hegele
David E. Heinrichs
Robert A. Heinzen
Antje Heit
Sophie Helaine
Andrew J. Henderson
Regine Hengge
Scott E. Hensley
Ute Hentschel
Aaron D. Hernday
Alfredo H. Herrera-Estrella
Dagmar Heuer
Erik L. Hewlett
J. Bernard Heymann
Meleah Hickman
Darren Higgins
Daniel F. Hoft
Deborah A. Hogan
Edward C. Holmes
Erik Holmqvist
Michael Hood
Alexander R. Horswill
Yilin Hu
Laura A. Hug
Philip Hugenholtz
Diarmaid Hughes
Kelly T. Hughes
Christina M. Hull
Christopher Hunter
Jason F. Huntley
Laurence Hurst
Aeron Christopher Hurt
Bonnie L. Hurwitz
Wilhelmina Huston
Jennifer Hyde
James Imlay
Keith Ireton
Ralph R. Isberg
Mary Ann Jabra-Rizk
William T. Jackson
William R. Jacobs, Jr.
Ilse D. Jacobsen
Ursula Jakob
Tim James
Guilhem Janbon
Dragana Jankovic
Mike S. Jetten
Marc C. Johnson
Nicola L. Jones
Diane Joseph-McCarthy
Gyoo Yeol Jung
Jae U. Jung
Pilar Junier
Mehdi Kabbage
David Kadosh
Jonathan Kagan
Martin Kaltenpoth
Hyun Ah Kang
Heidi B. Kaplan
Petr Karlovsky
Neerja Karnani
Wali Karzai
Fatah Kashanchi
Souichiro Kato
Andrew L. Kau
Angela Kaysel
Barbara I. Kazmierczak
Joseph Keane
Daniel B. Kearns
Patrick J. Kearns
Thomas E. Kehl-Fie
Bart J. F. Keijser
Scott T. Kelley
Felice D. Kelly
Stephen J. Kent
Nir Keren
Imtiaz Khan
M. Nadeem Khan
Patricia J. Kiley
Kami Kim
Paul R. Kinchington
David L. Kirchman
Kasper Urup Kjeldsen
Phillip E. Klebba
Jonas Klingstrom
Leigh Knodler
Michal Koblizek
Andrew Young Koh
Jay Kolls
Roberto Kolter
Anna Konovalova
Konstantinos T. Konstantinidis
Hyun Koo
Michael Koomey
Daniel Kornitzer
Vassilis Koronakis
Ákos T. Kovács
Kirsten J. Koymans
Thomas R. Kozel
Lukasz Kozubowski
Florian Krammer
Tino Krell
Eric J. Kremer
Vladimir Kren
Christopher J. Kristich
Thomas M. Kristie
Damian J. Krysan
Katharina Kubatzky
Meta Kuehn
Ed J. Kuijper
Ayush Kumar
Edo Kussell
Terry Kwok
Maurício Lacerda Nogueira
Laimonis A. Laimins
Seema Lakdawala
Michael Lalk
Tommy Tsan-Yuk Lam
Santo Landolfo
Morgan G. I. Langille
Ryan A. Langlois
Maria Lara-Tejero
Luis F. Larrondo
D. G. Joakim Larsson
Inigo Lasa
Matthew B. Lawrenz
Helen M. Lazear
Francois Lebreton
Bok Luel Lee
Jean Claire Lee
Margie D. Lee
Soo Chan Lee
Vincent T. Lee
Thomas Leitner
Jose Lemos
Mark Alexander Lever
Petra Anne Levin
Myron M. Levine
Daniel Lew
Kim Lewis
Dongmei Li
Hui-Ling Liao
Tine Rask Licht
Mark R. Liles
Brett D. Lindenbach
Paul Ling
Marc Lipsitch
Daniel Lipus
Haoping Liu
Nicolas Locker
Frank E. Loeffler
Volker Lohmann
Karl Lohner
Shee Mei Lok
Patrick Lomonte
Susana López
Jose L. Lopez-Ribot
Sebastian Lourido
Tiffany Marie Lowe-Power
Anice C. Lowen
Ling Lu
Francesca Luca
Carsten Lüder
Shan-Lu Lui
Aron E. Lukacher
Joseph Lutkenhaus
Erika I. Lutter
Zhonghua Ma
Jason Mackenzie
Gkikas Magiorkinis
Bernardo Alfredo Mainou
Shinji Makino
Jacob Malone
Balaji Manicassamy
Suliana Manley
Colin C. Manoil
John W. Mansfield
Michael D. Manson
Steven Mansoorabadi
Ernesto Marques
Luciano Marrafini
Jeanne Marrazzo
Mark Marsh
Jose L. Martinez
Thorsten Mascher
Paul S. Masters
Amy J. Mathers
Ivan Matic
Masao Matsuoka
Yu Matsuura
Karen L. Maxwell
Laura Mazzaferro
Shonna M. McBride
Honour McCann
Linda McCarter
John McCutcheon
Michael J. McInerney
Valerie McKenzie
James B. McKinlay
Rachel M. McLoughlin
Robert McMaster
Alan McNally
Joan Mecsas
Andrew Mehle
Roman Melnyk
Stephen B. Melville
Sabeeha S. Merchant
Stephane Meresse
Joris Messens
Thomas F. Meyer
Craig Meyers
Alessandro Michelucci
Jan Michiels
Aaron P. Mitchell
Masaki Miyake
Tomohiro Miyoshi
Harry L. T. Mobley
Edward S. Mocarski
Ian Mohr
Ian J. Molineux
Emmanuel F. Mongodin
David Montefiori
Cary A. Moody
Margo M. Moore
Nathaniel Moorman
Gary Moran
Iain M. Morgan
Julie Morrissey
Claus Moser
Chrystalla Mouza
Ryan Sean Mueller
Matthew A. Mulvey
Karl Münger
Vasant Muralidharan
Alicia M. Muro-Pastor
Erin R. Murphy
Timothy F. Murphy
Megan Murray
Lawrence C. Myers
D. Na
Julian R. Naglik
Francis E. Nano
Franz Narberhaus
Gioacchino Natoli
Ken Nealson
Sjaak Neefjes
Daniel C. Nelson
Eric Jorge Nelson
Jay Nelson
Martha I. Nelson
Mihai G. Netea
Benjamin W. Neuman
Michael M. Nevels
Irene L. G. Newton
Olivier Neyrolles
Minh Hong Nguyen
Frank Nichols
Kenneth Nickerson
Anthony V. Nicola
Jens Nielsen
Jean-Philippe Nougayrede
Mairi C. Noverr
Ole Nybroe
Spencer V. Nyholm
Christine M. O'Connor
Amanda G. Oglesby-Sherrouse
Thomas Vincent O'Halloran
Antonio Oliver
Erica Ollmann Saphire
Anders Omsland
Akira Ono
Ronald S. Oremland
Mary O'Riordan
William D. Orsi
Guadalupe Ortega-Pierres
Kim Orth
George O'Toole
Melanie Ott
Michael Otto
Malcolm G. P. Page
Gustavo Palacios
Mark John Pallen
Carla Palma
Bernhard O. Palsson
Timothy Palzkill
Kai Papenfort
Rebecca E. Parales
John S. Parkinson
Teresa E. Pawlowska
Rafael Peña-Miller
Richard M. Peek
Peter Pelka
José Penades
Miguel Penalva
Ines C. Pereira
Lena Pernas
Kevin Pethe
Britney Lee Phippen
Ted C. Pierson
Gorben Peter Pijlman
Lucia Pita
Johann Pitout
Javier Pizarro-Cerda
Vicente Planelles
Paul J. Planet
Pavel Plevka
Alexander Ploss
Kit Pogliano
Stefan H. Pöhlmann
Laurent Poirel
Patrice Polard
Michel-Robert Popoff
Owen Pornillos
Matteo Porotto
Evan Powers
Thomas Powers
Veena Prahlad
Michael B. Prentice
Gail M. Preston
Lance B. Price
Sean T Prigge
Alice S. Prince
Michael J. Pucci
Michael A. Purdy
Lotta Purkamo
Xiangguo Qiu
Cassandra L. Quave
Korneel Rabaey
Manuela Raffatellu
Tracy Lyn Raivio
Kumaran S. Ramamurthi
Juan L. Ramos
Glenn Randall
Philip N. Rather
Patrick C. Reading
Amit Reddi
Manjula Reddy
Stephen Reece
Michael Reese
Ryan Rego
Gemma Reguera
Jonathan S. Reichner
Larry Reitzer
Jyothi Rengarajan
Blanca I. Restrepo
Todd B. Reynolds
David Ribet
Andrew Rice
Charles Rice
Kelly C. Rice
Anthony R. Richardson
Sandra S. Richter
Bert K. Rima
Amariliz Rivera
Brian D. Robertson
David Robertson
Gregory Thomas Robertson
Eduardo Rocha
Alejandra Rodríguez-Verdugo
Andrew J. Roe
Richard John Roller
Thierry Rolling
Fabio Romerio
Ute Romling
David Rosen
Amy Rosenfeld
Ilan Rosenshine
Ramon Rosselló-Móra
Stefan Rothenburg
Kathrin Rousk
Brent J. Ryckman
Jeroen P. J. Saeij
Xavier Saelens
Sanjeev K. Sahni
George Sakoulas
Padmini Salgame
James E. Samuel
Álvaro San Millan
Alfonso Santos-Lopez
Carlos São-José
Saumendra N. Sarkar
Karla J. F. Satchell
John-Demian Sauer
Karin Sauer
Ram Savan
Jimmy Saw
Deepak Saxena
Claudio Scazzocchio
Luis M. Schang
Birgit E. Scharf
Mario Schelhaas
Mark A. Schembri
Michael Schindler
Simon Schmidt
Monika Schmoll
Dirk Schneider
John Schoggins
Herman B. Scholthof
Alexandra Schuetz
Maya Schuldiner
Julia Schumacher
Maria A. Schumacher
Martin Schuster
Martin Schwarzer
Peter Sebo
Frank Seeber
Anna Maria Seekatz
Anca M. Segall
Julia A. Segre
H. Steven Seifert
Adnane Sellam
Bert L. Semler
Jeremy D. Semrau
Allessandro Senes
Laura Serino
Ruth Serra-Moreno
Stephanie Seveau
Dionyssios N. Sgouras
Barbara Shacklett
Javeed Shah
Elizabeth Anne Shank
Jason W. Shapiro
Liam P. Shaw
Lindsey N. Shaw
Jessica R. Sheldon
Aimee Shen
Liang Shi
Pei-Yong Shi
Shuji Shigenobu
Patrick Shih
Joanna L. Shisler
Joshua D. Shrout
Rafael Silva-Rocha
Lynn L. Silver
Simon D. Silver
Lyle A. Simmons
Andrew C. Singer
Mitchell Singer
Upinder Singh
Varsha Singh
Photini Sinnis
Detmer Sipkema
Eric P. Skaar
James M. Slauch
Jason G. Smith
Joe Smith
Tara C. Smith
Lori Snyder
Shirleen Soh
Marc Solioz
David R. Soll
Jay V. Solnick
Abraham L. Sonenshein
Søren Johannes Sørensen
Victor Sourjik
Marie-Odile Soyer-Gobillard
Brad J. Spellberg
Tobias Spielmann
Alfred Michael Spormann
Eric V. Stabb
Jason E. Stajich
Richard Stanton
Lisa Y. Stein
William J. Steinbach
Noam Stern-Ginossar
Ann M. Stevens
Bradley Scott Stevenson
Frank J. Stewart
Philip S. Stewart
John F. Stolz
Brian D. Strahl
Paul D. Straight
Klaus Strebel
Daniel Streblow
Michel Strubin
Jorg Stulke
Lishan Su
Agathe Subtil
Joseph M. Suflita
William Sugden
Brian Martin Sullivan
William J. Sullivan
Baolin Sun
Nobuhiro Suzuki
Taichi Suzuki
Staffan G. Svärd
Mark Swain
Joel Swanson
W. Edward Swords
Michiko E. Taga
Fumihiro Taguchi
Andrew W. Tai
Nick Talbot
K. P. Connie Tam
Rita Tamayo
Pranita D. Tamma
Vera L. Tarakanova
Rick L. Tarleton
Neslihan Taş
Nuno Taveira
Gregory Taylor
Michael W. Taylor
Tracy Teal
Luis Teixeira
Benjamin R. tenOever
Ken Teter
Herve Tettelin
Rita Tewari
Michael G. Thomas
Vinai Chittezham Thomas
Isaac P. Thomsen
Anna D. Tischler
Peter Tontonoz
Janetta Top
Edward Topp
César I. Torres
Domenico Tortorella
Thierry Touzé
Matthew F. Traxler
Moritz Treeck
M. Stephen Trent
Kürsad Turgay
Anne-Catrin Uhlemann
Glen Ullett
Priya Uppuluri
Raphael Valdivia
Brian C. VanderVen
Marjan W. van der Woude
Gerlinde R. Van de Walle
Giel van Dooren
Julia C. van Kessel
Carine Van Lint
Laurence Van Melderen
Tim van Opijnen
Daria Van Tyne
Raghavan Varadarajan
Anna Vecchiarelli
Charlotte Vendrely
Ophelia Venturelli
Kevin Verstrepen
Valdimara C. Vieira
Adrien Vigneron
Patrick H. Viollier
Karen L. Visick
Michelle Visser
Marnix Vlot
Waldemar Vollmer
Veronika von Messling
Julia A. Vorholt
Ekaterina Voronina
Romé Voulhoux
Valmik K. Vyas
Slavena Vylkova
Gary E. Ward
John Wade
Samuel Wagner
Kevin John Waldron
Daniel Walker
David Walsh
Dai Wang
Fengping Wang
Jin-Town Wang
Jue D. Wang
Minggui Wang
Ping Wang
Qiuhong Wang
Shihua Wang
Yue Wang
Lewis Ward
Matthew J. Wargo
Martin J. Warren
Koichi Watashi
Andrew P. Waters
Christopher M. Waters
Kylie J. Watts
Scott C. Weaver
Richard J. Webby
Philipp Weimann
Daniel M. Weinberger
Daniel Weinreich
David S. Weiss
Matthew D. Weitzman
Rodney A. Welch
Sandra K. Weller
Melanie Wellington
Elizabeth A. White
John H. White
Judith M. White
Marvin Whiteley
Malcolm Whiteway
Chris Whitfield
Stephen Wikel
Annegret Wilde
Janet E. Williams
Brenda A. Wilson
Duncan W. Wilson
Emma H. Wilson
Patrick Wilson
Richard A. Wilson
Martin Witzenrath
Matthew C. Wolfgang
Gary Wong
Joshua J. Woodward
Floyd L. Wormley, Jr.
Roger Worrell
Daniel J. Wozniak
Rachel A. F. Wozniak
Brendan W. Wren
Gavin Wright
William Wuest
Slawomir Wybraniec
Karina B. Xavier
Jie Xiao
Jin-Rong Xu
Timothy L. Yahr
Hung-Chih Yang
Tomokazu Yoshinaga
Elton T. Young
Vincent B. Young
Hang Yu
Sung-Hwan Yun
Oscar Zaragoza
Raffaele Zarrilli
Fidel Zavala
Yan-Jin Zhang
Ying Zhang
Yongjie Zhang
Zhizhou Zhang
Yong-Hui Zheng
Rui Zhou
Jian Zhu
Pinkuan Zhu
Arturo Zychlinsky

